# A retinal origin of nystagmus—a perspective

**DOI:** 10.3389/fopht.2023.1186280

**Published:** 2023-06-08

**Authors:** Maarten Kamermans, Beerend H. J. Winkelman, M-B. Hölzel, Marcus H. C. Howlett, Wouter Kamermans, H. J. Simonsz, C. I. de Zeeuw

**Affiliations:** ^1^ Department of Retinal Signal Processing, Netherlands Institute for Neuroscience Amsterdam, Amsterdam, Netherlands; ^2^ Department of Biomedical Physics, Academic Medical Center, University of Amsterdam, Amsterdam, Netherlands; ^3^ Department of Cerebellum: Coordination & Cognition, Netherlands Institute for Neuroscience Amsterdam, Amsterdam, Netherlands; ^4^ Department of Neuroscience, Erasmus Medical Center, Rotterdam, Netherlands; ^5^ Department of Ophthalmology, Erasmus Medical Center, Rotterdam, Netherlands

**Keywords:** nystagmus, retinal ganglion cells, A_II_ amacrine cell, retina, accessory optic system (AOS)

## Abstract

Congenital nystagmus is a condition where the eyes of patients oscillate, mostly horizontally, with a frequency of between 2 and 10 Hz. Historically, nystagmus is believed to be caused by a maladaptation of the oculomotor system and is thus considered a disease of the brain stem. However, we have recently shown that congenital nystagmus associated with congenital stationary night blindness is caused by synchronously oscillating retinal ganglion cells. In this perspective article, we discuss how some details of nystagmus can be accounted for by the retinal mechanism we propose.

## Introduction

**Figure 1:**
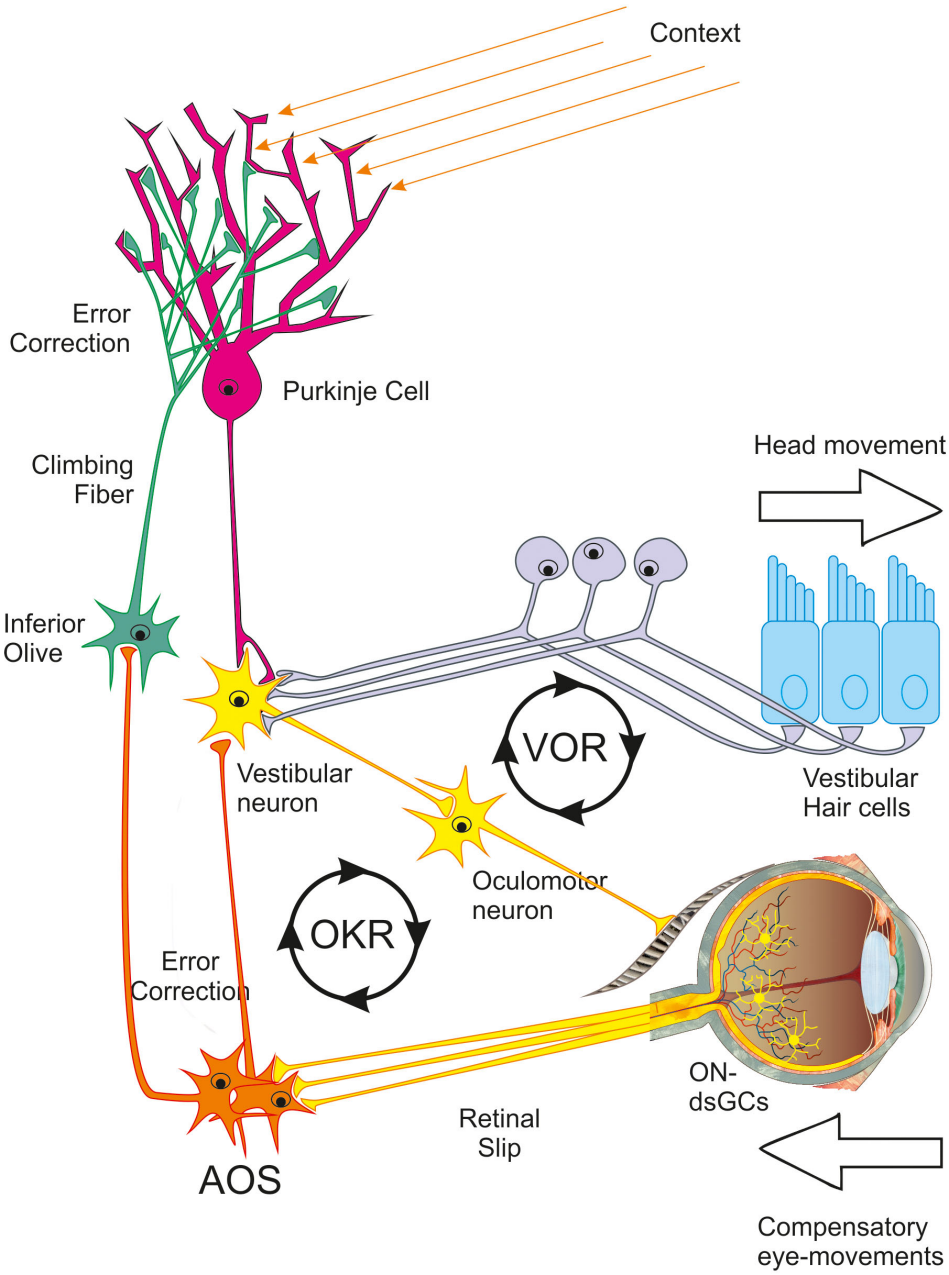
Schematic representation of the optokinetic reflex/vestibulo-ocular reflex system. For explanation see text. Modified figure by J. Pettigrew. OKR, optokinetic reflex; ON-dsGC, ON-direction-selective retinal ganglion cell; VOR, vestibulo-ocular reflex.

One of the most amazing abilities of the human brain is its ability to stabilize our sensory input while moving to facilitate perception. For example, we stabilize images on the retina while moving around in our environment. This is accomplished by close interactions between the sensory input from the retina and processing in the accessory optic system (AOS), the vestibulo-cerebellar system, and the oculomotor neurons that drive the eye muscles (**Figure 1**).

In general terms, the system works as follows: when we make a head movement, the vestibular hair cells detect this motion and drive the vestibular nucleus neurons. The oculomotor neurons, and eventually, the eye muscles then react, and compensatory eye movements occur. This loop is responsible for the vestibulo-ocular reflex (VOR). In addition, direction-selective circuits in the retina detect any remaining retinal image slip and send a signal *via* the ON-direction-selective retinal ganglion cells (ON-dsRGCs) to the AOS, the vestibular neurons, the oculomotor neurons, and, eventually, to the eye muscles such that this remaining image movement can be compensated for as well. This later loop is responsible for the so-called optokinetic reflex (OKR). When this mechanism malfunctions, nystagmus can be a consequence (1–4).

Nystagmus is characterized by involuntary, mostly horizontal, oscillating eye movements. There are many forms of nystagmus with many different causes and ages at onset. Nystagmus can be congenital (prevalence approximately 0.2% (5)**),** although, as it usually manifests sometime after birth, it is often called “infantile nystagmus”, or it can be acquired later in life as a result of neurological disorders (6). Typically, children with congenital nystagmus do not see an oscillating image, whereas adults with acquired nystagmus often perceive the visual field oscillating, i.e., oscillopsia.

## A plausible hypothesis for the cause of congenital nystagmus

**Figure 2:**
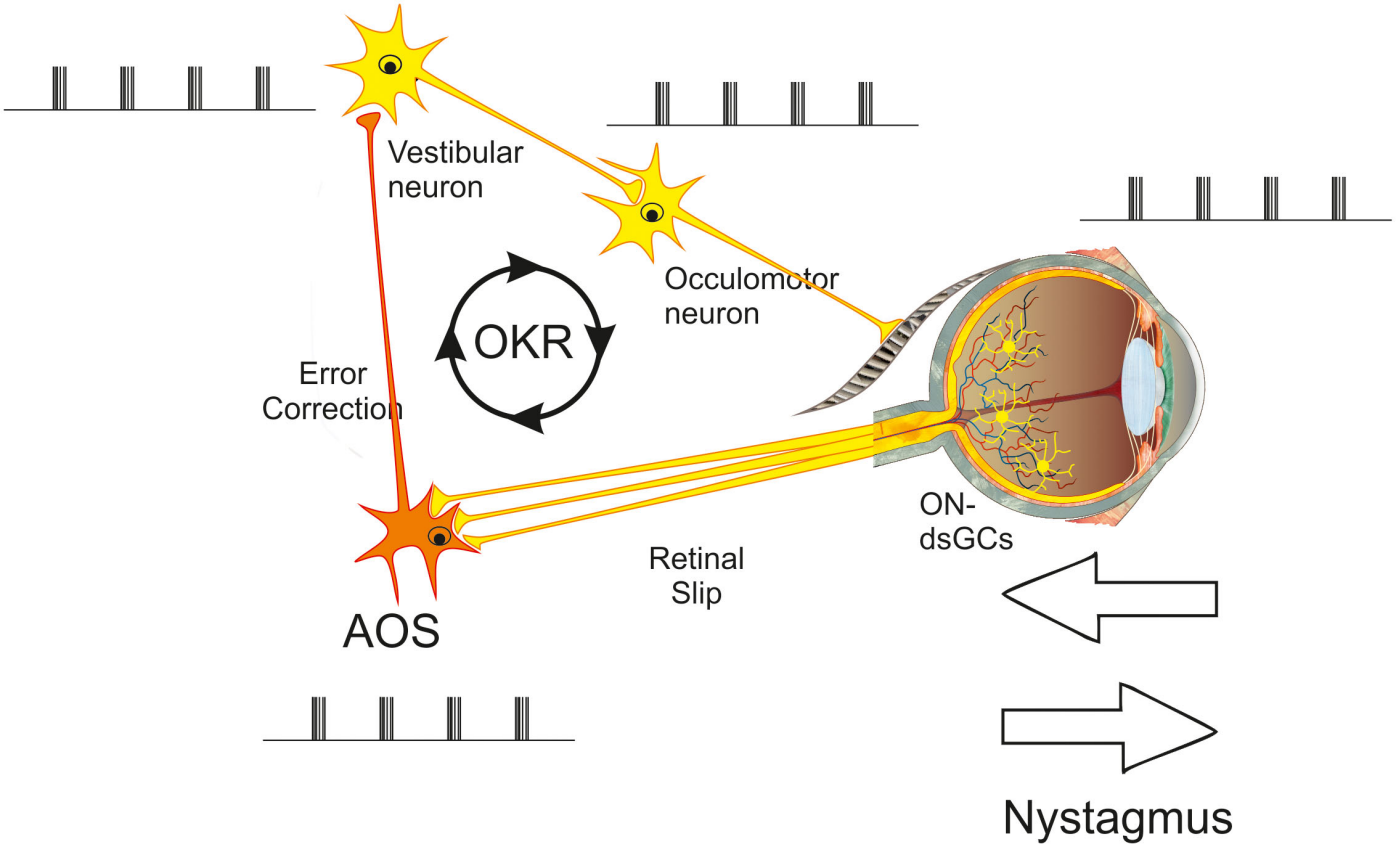
Example of an oscillating OKR loop. For explanation see text. AOS, accessory optic system; OKR, optokinetic reflex; ON-dsGC, ON-direction-selective retinal ganglion cell.

From a control systems perspective, the cause of congenital nystagmus could be simply a mistuned control loop. A simple feedback control loop with an integrator and a delay can oscillate because it can generate a sufficient phase shift for resonance to occur. When the gain is too high, this oscillation is not dampened, and the system will oscillate. For example, as the eye moves, the retinal image shifts, which is detected by the retinal circuit that detects global motion. The output of this system, the ON-dsRGCs, send a signal to the AOS (7–10), which is further relayed to the vestibular nucleus neurons and from there to the oculomotor neurons that drive the eye muscles, leading to a compensatory eye movement (**Figure 2**). Oscillatory eye movements can develop when the gain of this loop is too high or when a delay in this loop is too large (11, 12). If the signal induces a compensatory eye movement that is too large, it may generate additional global motion rather than compensate for it. This added (and erroneous) global motion signal will itself induce a new retinal signal for a compensatory eye movement, triggering the whole loop again and leading to nystagmus.

One can think of many variants of such a feedback control loop (see, for instance, Dell’Osso, 2006), but a common feature to all is that when congenital nystagmus is present, the oscillatory activity occurring throughout the OKR loop has the same frequency. Opening this oscillatory loop should stop the oscillations and the nystagmus.

**Figure 3:**
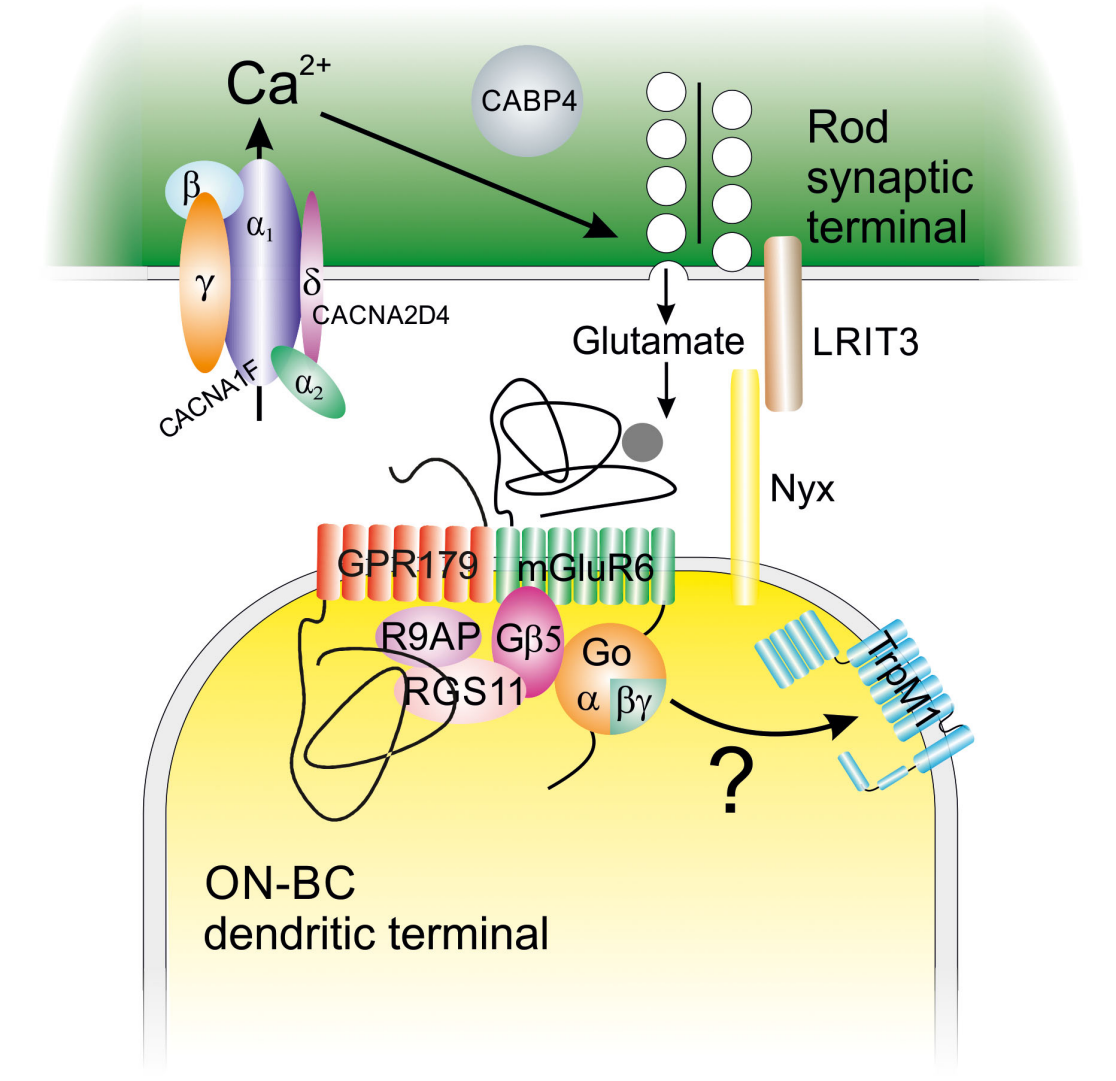
The metabotropic glutamatergic rod to ON-bipolar cell synapse with its molecular components. ON-BC, ON-bipolar cell.

However, we recently found evidence that the mechanism can be quite different (13). We studied *Nyx^nob^* mice, which suffer from a mutation in the gene encoding for nyctalopin, a protein located specifically at photoreceptor to ON-bipolar cell (BC) synapse (**Figure 3**). We found that these mice had a disturbed OKR response and a horizontal nystagmus of about 5 Hz. *In vivo* optic nerve recordings showed burst activity of about 5 Hz. So far, these results were consistent with an oscillating OKR loop. The surprise came when we isolated the retina and recorded retinal ganglion cell (RGC) spiking activity. The firing of RGCs in the isolated retina also oscillated with a frequency of about 5 Hz. This suggested a completely different origin of nystagmus: a retinal oscillator driving the oscillatory eye movements.

## Proof of causality

First, we tested whether or not the ON-dsRGCs were among the oscillating RGCs. SPIG1^+^ mice specifically express a green fluorescent protein (GFP) label in the ON-dsRGCs coding for upward motion. When we crossed the SPIG1^+^ mice with *Nyx^nob^* mice and recorded the activity of visually selected GFP-positive RGCs, these RGCs turned out to also oscillate in the isolated retina with a frequency of about 5 Hz (13). Next, we aimed to show the causality between the oscillating activity of ON-dsRGCs and the horizontal nystagmus. To show causality one needs to induce, block, and modify the RGC oscillations by retinal-specific interactions and show that the nystagmus changes in the same way.

### Induction of nystagmus

We studied three mouse models with mutated proteins in the photoreceptor to ON-BC synapse (i.e., *Nyx^nob^*, *mGluR6^–/–^*, and *Cacna1F^–/–^* mutants). These mutated proteins are virtually all retina specific and block the photoreceptor to ON-BC synapse. In all cases, we found that the horizontal nystagmus of the mice had the same frequency as that of the RGCs oscillations in the isolated retina (14).

### Block nystagmus

We could block the oscillations of RGCs in the isolated retina with a cocktail of 50 μM 6,7-dinitro-1,4-dihydroquinoxaline-2,3-dione (DNQX) and 10 μM “(2*S*)-2-amino-4-phosphonobutanoic (L-AP4). When we injected this cocktail in the eyes of *Nyx^nob^* mice, the nystagmus stopped (13). In principle, the DNQX/L-AP4 cocktail blocks the output of the retina, which in itself opens the OKR loop. Nystagmus could thus be absent because of the open loop or because of the block of input from a retinal oscillator to the AOS. To distinguish between these two conditions, we studied *Cacna1F^–/–^* mice. In these mice the photoreceptor output is fully blocked, making them effectively blind (14). This opens the OKR loop, just like the DNQX/L-AP4 cocktail, but at the input stage of the retina instead of at the output stage. Hence, the signal from a retinal oscillator can still reach the AOS and induce compensatory eye movements. Indeed, these mice had nystagmus (without vision), which shows that in these mice their nystagmus is driven by a retinal process and not by an oscillating OKR loop (14).

### Modify nystagmus

We could reduce the oscillation frequency of RGCs in the isolated retina from 5 to 2.5 Hz by adding 10 μM strychnine (STR) to the perfusate. Injecting STR into the eyes of *Nyx^nob^* mice led to a similar reduction in the oscillation frequency of the nystagmus (13).

These experiments prove that there is a causal relationship between the oscillations of RGCs and the oscillatory eye movements in the mice mutants highlighted above. The oscillating RGC activity by itself drives the nystagmus, independent of processes intrinsic to the OKR loop downstream. But what exactly is oscillating in the retina? To answer that question, we need to dive deeper into the retinal physiology.

## The oscillator

The photoreceptor to ON-BC synapse is a unique sign-inverting glutamatergic synapse. When photoreceptors depolarize, their L-type calcium (Ca) channels (*Cacna1F*) open and Ca^2+^ flows into the photoreceptor and induces glutamate release. Glutamate diffuses across the synaptic cleft and activates the metabotropic glutamate receptor mGluR6 on the ON-BC dendrites. This initiates an intracellular cascade that eventually closes the *TRPM1* channel, which hyperpolarizes the ON-BC (**Figure 3**) (15–19). All the post-synaptic proteins involved are localized to the ON-BC dendrite by nyctalopin (Nyx). Nyctalopin expression requires the expression of the presynaptic protein *Lrit3* (20). Mutations in *Cacna1F*, *Lrit3*, mGluR6, *TRPM1*, *Gpr*179, or Nyx eliminate synapse function and light-evoked responses of ON-BCs (18, 19, 21, 22). Furthermore, patients and mice with these mutations suffer from congenital stationary night blindness (CSNB).

The glutamate released by the photoreceptors closes a conductance in the ON-BC, with a reversal potential of around 0 mV leading to hyperpolarization of the ON-BC. Hence, blocking the glutamate release or removing the metabotropic glutamate receptors is expected to depolarize the ON-BCs. When ON-BCs depolarize, A_II_ amacrine cells (ACs) will also depolarize as they receive glutamatergic input from rod-driven ON-BCs and are electrically coupled to cone-driven ON-BCs (23). In addition, either directly or indirectly, the A_II_ ACs also receive a crossover inhibitory signal from the OFF-pathway such that light stimulation induces depolarization of the A_II_ ACs (24).

**Figure 4:**
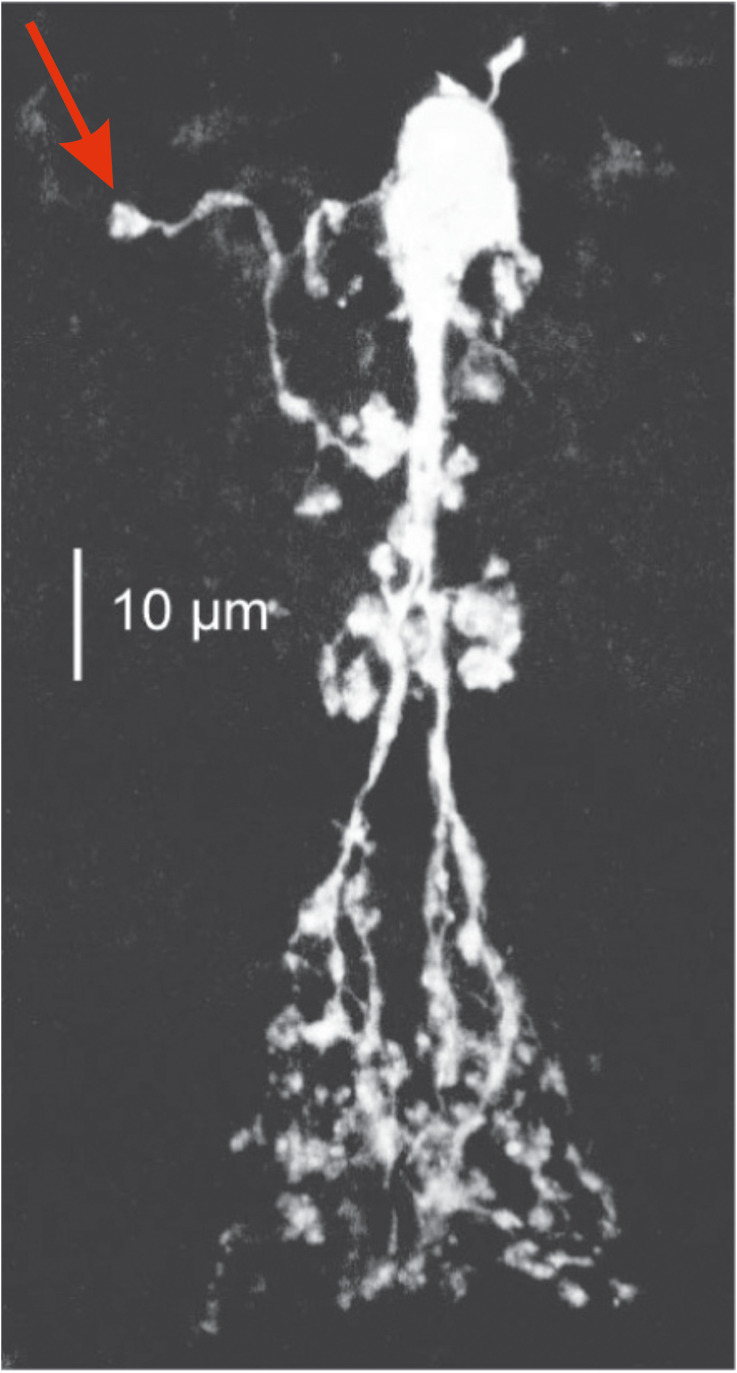
A dye-filled A_II_ AC with its initiation site (red arrow) Taken from Choi et al., 2014. AC, amacrine cell.

A_II_ ACs are an essential part of the primary rod pathway, relaying the signals from the rod-driven ON-BCs to the cone-driven ON- and OFF-BCs. The A_II_ ACs express a number of voltage-gated channels, including fast A-type and slow M-type K^+^, and fast Na^+^ channels (25). These channels are localized to a highly specialized A_II_ AC region: the initiation site, which is located on a long process sprouting from the A_II_ AC soma. The process itself does not contact any other cell and expresses these voltage-gated ion channels only at its tip (**Figure 4**).

This setup constitutes an active element in the A_II_ ACs that amplifies rod-driven, single-photon responses under scotopic conditions (26) and speeds up rod-driven responses under mesopic conditions. However, under pathological conditions where the A_II_ ACs are strongly depolarized, these voltage-gated ion-channels at the initiation site can lead to oscillations. This happens in the mouse model for retinitis pigmentosa, where the A_II_ AC membrane potential oscillates with a frequency of about 10 Hz. In addition, Choi and colleagues (25) have shown that, depending on the A_II_ ACs membrane potential, the oscillation frequency can vary between 2 and 10 Hz.

To test whether or not A_II_ ACs also generate the oscillations in the CSNB mouse models we recorded A_II_ ACs from isolated retinas and found that they indeed oscillate (unpublished results). Next, we pharmacologically changed their oscillation frequency by applying 30 μM linopirdine hydrochloride (LP), a blocker of M-type K^+^ channels, to the isolated retina or injected it into the eyes of mice. LP application reduced the oscillation frequency of RGCs in the isolated retina and the frequency of the nystagmus recorded *in vivo* to the same extent (13). 

**Figure 5:**
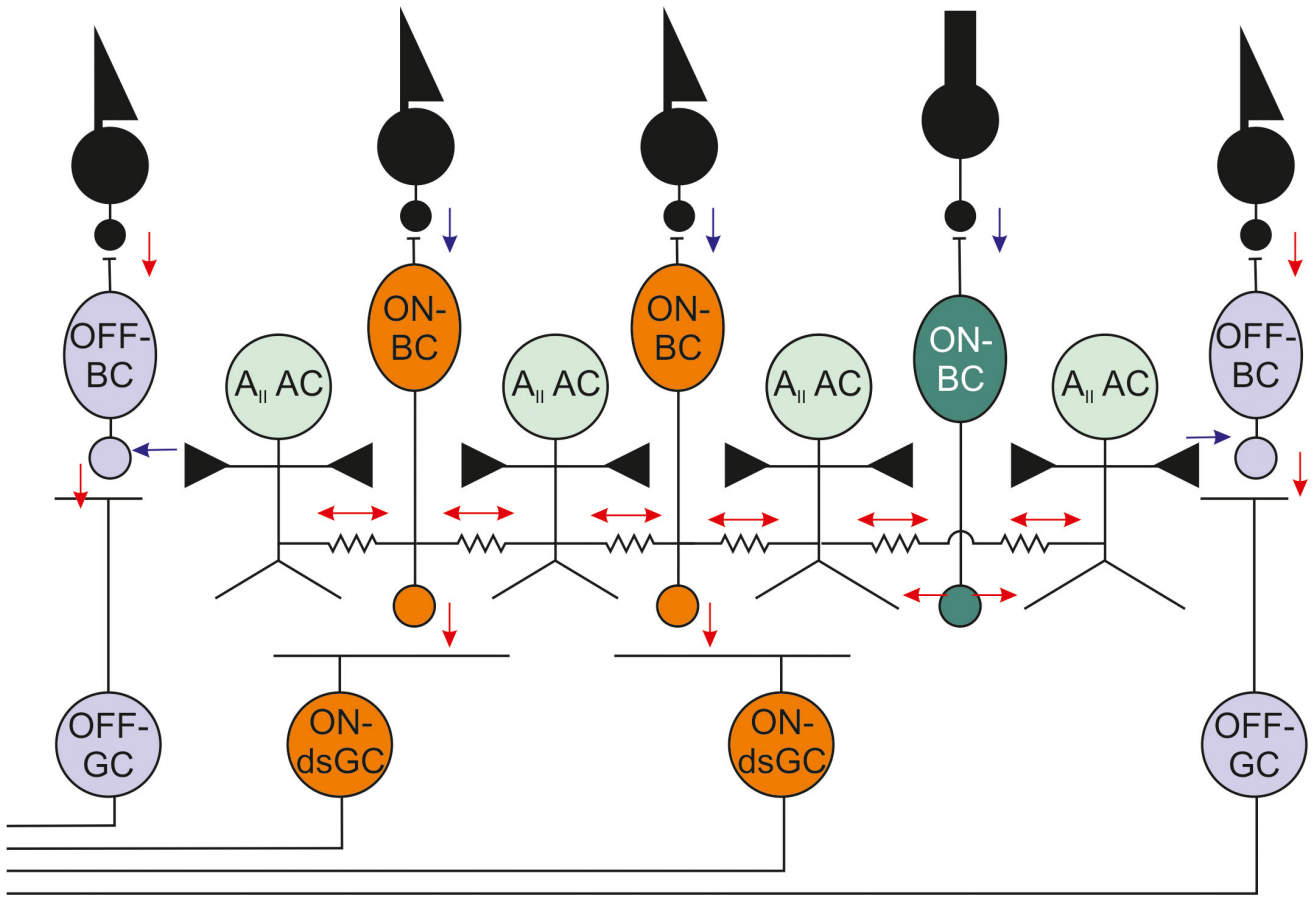
Schematic of the A_II_ AC network with its main inputs and outputs. AC, amacrine cell; BC, bipolar cell; dsGC, direction-selective ganglion cell; GC, ganglion cell.

The output of the A_II_ ACs affects ON-dsRGCs *via* the electrical coupling of the A_II_ ACs with the cone-driven ON-BCs (**Figure 5**). These gap junctions consist of Cx45 at the ON-BC side and Cx36 at the A_II_ AC side. Blocking the gap junctions pharmacologically or removing Cx36 should prevent the A_II_ ACs oscillations from entering the retinal circuit and inducing oscillations in RGCs, in effect stopping the nystagmus. Indeed, when we crossed the *Nyx^nob^* mice with Cx36^–/–^ mice we found that the RGCs were not oscillating and that the 5-Hz nystagmus was absent. These pharmacological and genetic experiments identify the A_II_ ACs as the critical source of the oscillations.

## The mechanism

Nystagmus in the *NYX^nob^* mice is absent in the dark and becomes more vivid in strong light conditions. What is the underlying mechanism for this light dependency of nystagmus? First, we tested whether or not the RGCs oscillated in the dark. To our surprise, the RGCs oscillated even though nystagmus was absent in this condition. Scrutinizing the results revealed that the oscillations of the different RGCs in the dark all had different frequencies and phases. However, light stimulation led to a unifying phase reset of the oscillations, resulting in synchronized RGC oscillations (13).

The AOS receives input from many ON-dsRGCs, which it uses to determine whether or not there is global image motion. When RGCs are oscillating out of phase with different frequencies, as occurs in the dark, the integrated ON-dsRGC signal in the AOS will be very small. Only when the RGCs oscillations are synchronized by a phase reset, as occurs after light stimulation, does the integrated signal in the AOS becomes large enough to induce a compensatory eye movement. Moreover, as the RGCs oscillate, this compensatory eye movement will be an oscillating compensatory eye movement, i.e., a nystagmus.

## Development and adaptation

So far, we have described the mechanism inducing nystagmus as purely retinal. However, secondary changes in the OKR/VOR system could also contribute. During development, the phases and gains of the OKR and VOR are tuned such that we can optimally stabilize our eyes when moving our head. This tuning depends to a large extent on direction information received from the ON-dsRGCs. In the *NYX^nob^* mice, this information is not available because the ON-pathway is not functional. As a consequence, the OKR/VOR system does not develop properly and the gains in the loop may become too high (Winkelman et al., 2019), which could lead to additional entrainment of the retinal oscillations which may increase the nystagmus.

In some CSNB mouse models only the ON-pathway is affected, whereas for others the OFF-pathway can be affected as well (20, 27). Thus, depending on the type of CSNB, some direction-selective information may still be available *via* the ON/OFF-dsRGCs. These cells project to the visual cortex (28, 29) and are involved in local motion detection. For mutations affecting only the ON-pathway, the ON/OFF-dsRGCs will, *via* their intact OFF-response, still send some direction information to the visual cortex. From there it will eventually be relayed to the AOS system and induce some adaptation of the OKR/VOR system. As the various mutations causing CSNB affect the ON- and OFF-pathways to different extents, subtle differences in OKR, VOR, and the waveform of the nystagmus are to be expected.

Thus, the different oscillation frequencies of the A_II_ ACs and all sorts of secondary effects may very well form the basis of the large variety of waveforms of nystagmus.

## Patients’ experiences

It has long been known that congenital nystagmus intensifies with increasing light intensity, especially when a visual task is requested, for instance when measuring visual acuity. Can we understand this aspect of nystagmus in the context of the retinal mechanism as well? It is to be expected that increasing light intensity will lead to a further depolarization of the A_II_ ACs. A_II_ ACs that are more strongly depolarized will oscillate with a higher frequency. Indeed, the oscillation frequency of RGCs when stimulated with dim light is lower than when stimulated with bright light (14). Thus, the retinal mechanism we propose is at least consistent with the association between light and nystagmus intensity observed in patients.

In contrast to patients with acquired nystagmus, patients with congenital nystagmus almost never complain of experiencing oscillating images, i.e., oscillopsia. Only when the velocity of the nystagmus is high, mostly in young people, is a flickering light sensation perceived (30). This subjective experience is in line with the oscillations of RGCs. So far, we have discussed the role of ON-dsRGCs, but they were only one of the many RGC types we found oscillating in the retinas of CSNB mouse models. Many of these other RGC types project to brain areas involved in visual perception. It is conceivable that, when these RGCs oscillate, this signal is translated into the perception of a flickering image.

## Is congenital nystagmus in other diseases also caused by oscillations in the retina?

Oscillating A_II_ ACs cause nystagmus in CSNB. In the examples discussed so far, the A_II_ ACs are oscillating because they are in a condition of sustained depolarization owing to the mutated photoreceptor to ON-BC synaptic proteins. The question arises whether or not nystagmus also occurs in other conditions where A_II_ ACs are strongly depolarized.

A condition where A_II_ ACs are strongly depolarized is retinitis pigmentosa (RP). Choi and colleagues (25) showed that A_II_ ACs oscillate in the retinal degeneration 1 (rd1) mouse, but nystagmus has not yet been demonstrated in these mice. In human RP patients, nystagmus is reported in some patients but not in others (31, 32). How can this diversity be explained? For the nystagmus we studied, two conditions had to be met: (1) A_II_ ACs need to oscillate, and (2) the oscillation must be phase reset by a light stimulus, i.e., the oscillations are synchronized by a contrast edge moving over the retina. In RP patients, the A_II_ ACs and RGCs are oscillating too (25). However, RP patients lose their peripheral vision and the ON-dsRGCs detecting global motion are present mostly in the peripheral retina. Hence, in advanced stages of RP it seems possible that light stimulation is no longer able to synchronize the ON-dsRGCs and so nystagmus may be absent as a result of this. In the early developmental stages of RP, there may be a time window when the A_II_ ACs are already oscillating but the light sensitivity has not yet deteriorated severely. In this case, the remaining light sensitivity may still be sufficient to phase reset the A_II_ AC oscillations and induce nystagmus.

Recently a specific mutation in Munc18 (mammalian uncoordinated)-18 was reported to lead to nystagmus (33). The mutation leading to nystagmus enhances the binding of Munc18 with syntaxin3B. The Munc18–syntaxin complex is essential for docking synaptic vesicles at the active zone of a synapse. Almost all synapses use syntaxin1A, except photoreceptors, which use syntaxin3B. A likely scenario in this case is that this specific Munc18 mutation is functionally affecting only photoreceptor synaptic transmission leading to depolarization of the ON-BCs and A_II_ ACs. As a result, the A_II_ ACs will start to oscillate and nystagmus appears, whereas the same Munc18 mutation leaves other synapses unaffected.

A_II_ ACs are highly interconnected neurons. They receive at least glutamatergic, GABAergic, glycinergic, and dopaminergic input. Disturbances in these inputs may cause the A_II_ AC to depolarize, resulting in nystagmus. Future genetic studies into the causes of nystagmus should at least consider the possibility that the mutation identified may have affected the A_II_ ACs inputs and in that way have induced nystagmus.

## Is this system also functional in the primate retina?

It has long been argued that in primates dsRGCs are absent in the retina [see, for instance, Bach, and Hoffmann (34), and, instead, direction information is calculated in the visual cortex. Indeed, until recently, there was no evidence for dsRGCs in primates. However, recent studies show direct evidence of the presence of ON-dsRGCs in the primate retina (Puthussery et al., in press, https://www.youtube.com/watch?v=VqGGBOsOA-c). Why did it take so long to find these RGCs? A possible explanation for this is that the primate retina developed a fovea, which led to an enormous increase in alpha and parasol RGCs. Consequently, the fraction of RGCs selective for direction becomes very small. Indeed, only 1%–2.5% of the RGCs seem to be ON-dsRGCs. However, this does not mean that they are less important. Rather, it may indicate that far fewer cells are needed to detect global motion than for, say, high spatial acuity.

## Conclusion

Nystagmus in CSNB is caused by a retinal oscillator: the A_II_ ACs. These cells drive oscillations in ON-dsRGCs, which in turn project to the AOS where they induce “compensatory” eye movements. These eye movements can be further enhanced/modified by secondary adaptations in the OKR loop. A_II_ ACs start to oscillate when their membrane potential is depolarized outside its normal operating range. As A_II_ ACs are highly interconnected neurons, it is possible that many disease conditions may affect their membrane potential. Hence, the mechanism we have described underlying nystagmus in CSNB also has the potential to cause nystagmus in many other eye conditions. Therefore, considering that nystagmus may have a retinal origin, it is crucial to advance congenital nystagmus research under a wider variety of genetic and environmental conditions.

## Data availability statement

Publicly available datasets were analyzed in this study. These data can be found here: https://figshare.com/account/home#/projects/65990.

## Ethics statement

All animal experiments were carried out under the responsibility of the ethics committee of the Royal Netherlands Academy of Arts and Sciences (KNAW) acting in accordance with the European Communities Council Directive of 22 July 2003 (2003/65/CE). The experiments were performed under the license number AVD-801002016517, issued by the Central Committee Animal Experiments of the Netherlands.

## Author contributions

All authors contributed to the article and approved the submitted version.

## References

[1] Panthagani J Virdee J MacDonald T Bruynseels A Batra R. Acquired nystagmus. Br J Hosp Med (Lond) (2020) 81:1–8. doi: 10.12968/hmed.2020.0320 33263469

[2] Strupp ML Straumann D Helmchen C. Central ocular motor disorders: clinical and topographic anatomical diagnosis, syndromes and underlying diseases. Klinische Monatsblatter fur Augenheilkunde (2021) 238:1197–211. doi: 10.1055/a-1654-0632 34784643

[3] Strupp ML Straumann D Helmchen C. Nystagmus: diagnosis, topographic anatomical localization and therapy. Klinische Monatsblatter fur Augenheilkunde (2021) 238:1186–95. doi: 10.1055/a-1525-0030 34784642

[4] Strupp M Kremmyda O Adamczyk C Bottcher N Muth C Yip CW. Central ocular motor disorders, including gaze palsy and nystagmus. J Neurol (2014) 261 Suppl 2:S542–58. doi: 10.1007/s00415-014-7385-9 PMC4141156 25145891

[5] Sarvananthan N Surendran M Roberts EO Jain S Thomas S Shah N. The prevalence of nystagmus: the Leicestershire nystagmus survey. Invest Ophthalmol Vis Sci (2009) 50:5201–6. doi: 10.1167/iovs.09-3486 19458336

[6] Papageorgiou E McLean RJ Gottlob I. Nystagmus in childhood. Pediatr Neonatol (2014) 55:341–51. doi: 10.1016/j.pedneo.2014.02.007 25086850

[7] Giolli RA Blanks RH Lui F. The accessory optic system: basic organization with an update on connectivity, neurochemistry, and function. Prog.Brain Res (2006) 151:407–40. doi: 10.1016/S0079-6123(05)51013-6 16221596

[8] Oyster CW Simpson JI Takahashi ES Soodak RE. Retinal ganglion cells projecting to the rabbit accessory optic system. J Comp Neurol (1980) 190:49–61. doi: 10.1002/cne.901900105 7381054

[9] Dhande OS Estevez ME Quattrochi LE El-Danaf RN Nguyen PL Berson DM. Genetic dissection of retinal inputs to brainstem nuclei controlling image stabilization. J Neurosci (2013) 33:17797–813. doi: 10.1523/JNEUROSCI.2778-13.2013 PMC3818553 24198370

[10] Oyster CW Takahashi E Collewijn H. Direction-selective retinal ganglion cells and control of optokinetic nystagmus in the rabbit. Vision Res (1972) 12:183–93. doi: 10.1016/0042-6989(72)90110-1 5033683

[11] Schweigart G Mergner T Evdokimidis I Morand S Becker W. Gaze stabilization by optokinetic reflex (OKR) and vestibulo-ocular reflex (VOR) during active head rotation in man. Vision Res (1997) 37:1643–52. doi: 10.1016/S0042-6989(96)00315-X 9231230

[12] Averbuch-Heller L Zivotofsky AZ Das VE DiScenna AO Leigh RJ. Investigations of the pathogenesis of acquired pendular nystagmus. Brain (1995) 118(Pt 2):369–78. doi: 10.1093/brain/118.2.369 7735879

[13] Winkelman BHJ Howlett MHC Holzel MB Joling C Fransen KH Pangeni G. Nystagmus in patients with congenital stationary night blindness (CSNB) originates from synchronously firing retinal ganglion cells. PloS Biol (2019) 17:e3000174. doi: 10.1371/journal.pbio.3000174 31513577 PMC6741852

[14] Holzel M-B Winkelman B Howlett MHC Kamermans W De Zeeuw CI Kamermans M. A common cause for nystagmus in different congenital stationary night blindness mouse models. BioRxiv (2023). doi: 10.1101/2023.04.24.538135 37864560

[15] Ray TA Hasan N McCall MA Tosini G Nishina PM Peachey NS. GPR179, an orphan G protein-coupled receptor, is critical to depolarizing cell function and interacts with GRM6. ARVO Abstracts (2012) 2012:3156.

[16] Morgans CW Bayley PR Oesch NW Ren G Akileswaran L Taylor WR. Photoreceptor calcium channels: insight from night blindness. Vis Neurosci (2005) 22:561–8. doi: 10.1017/S0952523805225038 16332266

[17] Gregg RG Mukhopadhyay S Candille SI Ball SL Pardue MT McCall MA. Identification of the gene and the mutation responsible for the mouse nob phenotype. Invest Ophthalmol Vis Sci (2003) 44:378–84. doi: 10.1167/iovs.02-0501 12506099

[18] Gregg RG Kamermans M Klooster J Lukasiewicz PD Peachey NS Vessey KA. Nyctalopin expression in retinal bipolar cells restores visual function in a mouse model of complete X-linked congenital stationary night blindness. J Neurophysiol (2007) 98:3023–33. doi: 10.1152/jn.00608.2007 PMC2933657 17881478

[19] Pardue MT McCall MA LaVail MM Gregg RG Peachey NS. A naturally occurring mouse model of X-linked congenital stationary night blindness. Invest Ophthalmol Vis Sci (1998) 39:2443–9. 9804152

[20] Hasan N Pangeni G Ray TA Fransen KM Noel J Borghuis BG. LRIT3 is required for nyctalopin expression and normal ON and OFF pathway signaling in the retina. eNeuro (2020) 7(1):0002–20. doi: 10.1523/ENEURO.0002-20.2020 PMC7031853 31959619

[21] Demas J Sagdullaev BT Green E Jaubert-Miazza L McCall MA Gregg RG. Failure to maintain eye-specific segregation in nob, a mutant with abnormally patterned retinal activity. Neuron (2006) 50:247–59. doi: 10.1016/j.neuron.2006.03.033 16630836

[22] Scalabrino ML Boye SL Fransen KM Noel JM Dyka FM Min SH. Intravitreal delivery of a novel AAV vector targets ON bipolar cells and restores visual function in a mouse model of complete congenital stationary night blindness. Hum Mol Genet (2015) 24:6229–39. doi: 10.1093/hmg/ddv341 PMC4612567 26310623

[23] Marc RE Anderson JR Jones BW Sigulinsky CL Lauritzen JS. The all amacrine cell connectome: a dense network hub. Front Neural circuits (2014) 8. doi: 10.3389/fncir.2014.00104 PMC4154443 25237297

[24] Werblin FS. Six different roles for crossover inhibition in the retina: correcting the nonlinearities of synaptic transmission. Vis.Neurosci (2010) 27:1–8. doi: 10.1017/S0952523810000076 20392301 PMC2990954

[25] Choi H Zhang L Cembrowski MS Sabottke CF Markowitz AL Butts DA. Intrinsic bursting of AII amacrine cells underlies oscillations in the rd1 mouse retina. J Neurophysiol (2014) 112:1491–504. doi: 10.1152/jn.00437.2014 PMC4137253 25008417

[26] Smith RG Vardi N. Simulation of the AII amacrine cell of mammalian retina: functional consequences of electrical coupling and regenerative membrane properties. Visual Neurosci (1995) 12:851–60. doi: 10.1017/S095252380000941X 8924409

[27] Zeitz C Robson AG Audo I. Congenital stationary night blindness: an analysis and update of genotype-phenotype correlations and pathogenic mechanisms. Prog Retin Eye Res (2015) 45:58–110. doi: 10.1016/j.preteyeres.2014.09.001 25307992

[28] Cruz-Martin A El-Danaf RN Osakada F Sriram B Dhande OS Nguyen PL. A dedicated circuit links direction-selective retinal ganglion cells to the primary visual cortex. Nature (2014) 507:358–61. doi: 10.1038/nature12989 PMC4143386 24572358

[29] Liu BH Huberman AD Scanziani M. Cortico-fugal output from visual cortex promotes plasticity of innate motor behaviour. Nature (2016) 538:383–7. doi: 10.1038/nature19818 PMC5460756 27732573

[30] Tusa RJ Zee DS Hain TC Simonsz HJ. Voluntary control of congenital nystagmus. Clin Vision Sci (1992) 7:195–210.

[31] Krill AE Deutman AF Fishman M. The cone degenerations. Documenta ophthalmologica (1973) 35:1–80. doi: 10.1007/BF00234530 4573331

[32] Tsang SH Sharma T. Leber congenital amaurosis. Adv Exp Med Biol (2018) 1085:131–7. doi: 10.1007/978-3-319-95046-4_26 30578499

[33] Li Y Jiang L Wang L Wang C Liu C Guo A. p.His16Arg of STXBP1 (MUNC18-1) associated with syntaxin 3B causes autosomal dominant congenital nystagmus. Front Cell Dev Biol (2020) 8:591781. doi: 10.3389/fcell.2020.591781 33251218 PMC7672047

[34] Bach M Hoffmann MB. Visual motion detection in man is governed by non-retinal mechanisms. Vision Res (2000) 40:2379–85. doi: 10.1016/S0042-6989(00)00106-1 10915879

